# Supplementary material for: Venomix: a simple bioinformatic pipeline for identifying and characterizing toxin gene candidates from transcriptomic data
Source: PeerJ. 2018 Jul 31;6:e5361. doi: 10.7717/peerj.5361 (PMC6074769; doi:10.7717/peerj.5361)
Supplement: Supplemental Information 6 [file peerj-06-5361-s006.gz › FinalOutput_1E-6/Venom_allergen_5_1/finaltree.pdf]

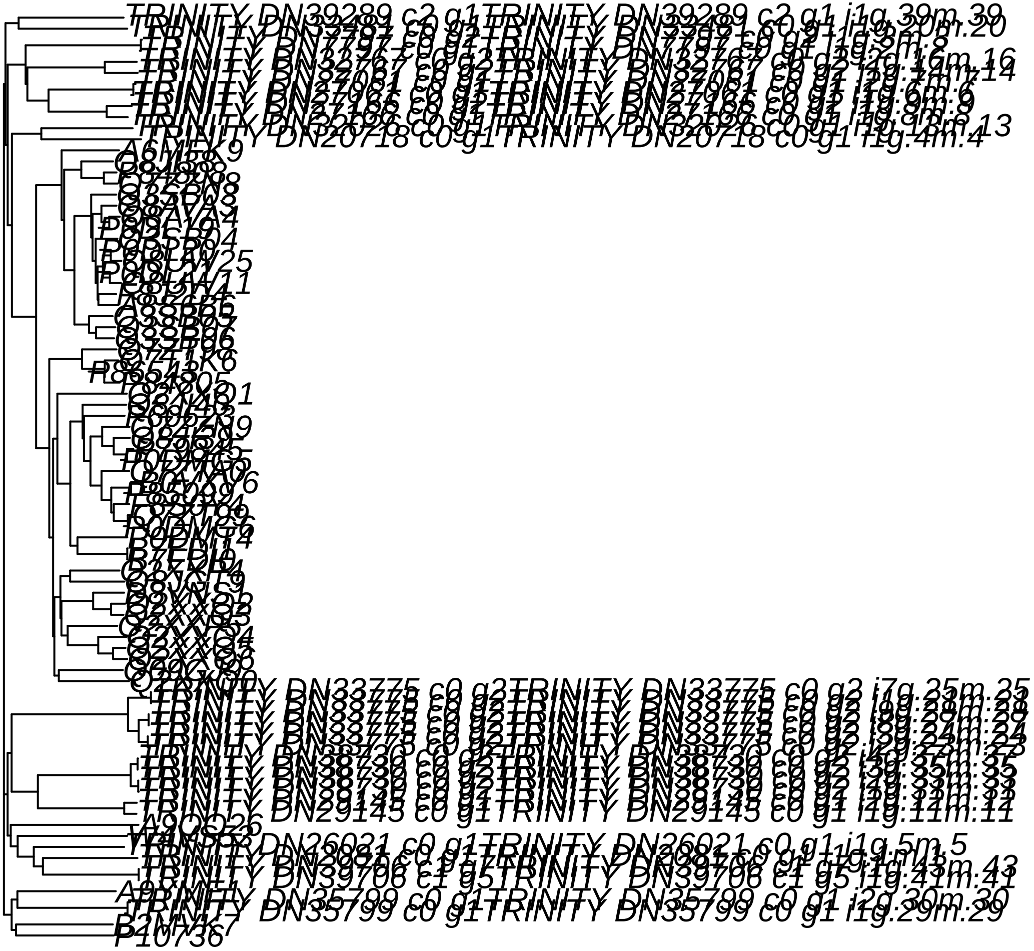
TRINITY DN39289 c2 g1TRINITY DN39289 c2 g1 i1g.39m.39
TRINITY DN20718 c0 g1TRINITY DN20718 c0 g1 i1g.4m.4
A6MLK9
TRINITY DN33775 c0 g2TRINITY DN33775 c0 g2 i7g.25m.25
TRINITY DN29145 c0 g1TRINITY DN29145 c0 g1 i1g.11m.11
A9QQ26
TRINITY DN26021 c0 g1TRINITY DN26021 c0 g1 i1g.5m.5
TRINITY DN39706 c1 g5TRINITY DN39706 c1 g5 i1g.41m.41
TRINITY DN35799 c0 g1TRINITY DN35799 c0 g1 i2g.30m.30
TRINITY DN35799 c0 g1TRINITY DN35799 c0 g1 i1g.29m.29
P10736
